# Temporary Passive Shunt for Visceral Protection During Open Thoracoabdominal Aortic Repair Under Intraoperative Advanced Hemodynamic and Perfusion Monitoring: Tertiary Hospital Institutional Bundle and Preliminary Mid-Term Results

**DOI:** 10.3390/jcm14176064

**Published:** 2025-08-27

**Authors:** Ottavia Borghese, Marta Minucci, Elena Jacchia, Pierfrancesco Antonio Annuvolo, Lucia Scurto, Antonio Luparelli, Andrea Russo, Paola Aceto, Tommaso Donati, Yamume Tshomba

**Affiliations:** 1 Unit of Vascular Surgery, Fondazione Policlinico Universitario A. Gemelli I.R.C.C.S, 00168 Rome, Italy; marta.minucci@unicatt.it (M.M.); elena.jacchia@unicatt.it (E.J.); pierfrancescoantonio.annuvolo@unicatt.it (P.A.A.); lucia.scurto@unicatt.it (L.S.); antonio.luparelli@guest.policlicnicogemelli.it (A.L.); tommaso.donati@policlicnicogemelli.it (T.D.); yamume.tshomba@policlinicogemelli.it (Y.T.); 2 Department of Cardiovascular Sciences, Vascular and Endovascular Surgery School, Faculty of Medicine and Surgery, Università Cattolica del Sacro Cuore, 00168 Rome, Italy; andrea.russo@policlicnicogemelli.it (A.R.); paola.aceto@policlinicogemelli.it (P.A.); 3 Department of Emergency, Anaesthesiologic and Reanimation Sciences, Fondazione Policlinico Universitario, A. Gemelli I.R.C.C.S., 00168 Rome, Italy

**Keywords:** Thoraco-Abdominal Aneurysm Repair, TAAA, complications, end-organ ischemia, visceral protection, renal failure, paraplegia, paraparesis

## Abstract

**Background**: The perfusion of viscera, kidney, and spinal cord represents one of the main concerns during open repair (OR) of Thoraco-Abdominal Aortic Aneurisms (TAAAs). Passive shunting (PS) has been historically used for intraoperative distal aortic perfusion but has been progressively replaced almost entirely by partial left-sided heart or total cardiopulmonary bypass with extra-corporeal circulation (ECC). Despite several advantages of these methods, PS still has potential in mitigating some drawbacks of long extracorporeal circuits connected with centrifugal or roller pumps, such as the need for cardiac and great vessels cannulation, priming and large intravascular fluid volume shifts, high heparin dose, immunosuppressive effects, and systemic inflammatory response syndrome. **Methods**: This study prospectively analyzed data of a cohort of patients who underwent TAAA OR using a PS in a single institution. Outcomes of interest were mortality, rate of mesenteric, renal and spinal cord ischemia, cardiac complications, and intraoperative hemodynamic stability achieved in this setting. Our institutional bundle and a comprehensive literature review about the different configurations and applicability of PS for TAAA OR is also reported. The search was performed based on three databases (PubMed, EMBASE, and Cochrane Library) by two independent reviewers (LS and AA) from inception to 31 December 2023, and the reported clinical results (visceral, renal, and spinal cord complications and mortality) using PS during TAAAs OR were analyzed. **Results**: Between March 2021 and December 2023, 51 TAAA repairs were performed and eleven patients (n = 8, 73% male; mean age 67 years, range 63–79) were operated using a PS for a total of one (9%) type I, one (9%) type II, two (18%) type III, five (45%) type IV, and two (18%) type V TAAA. In our early experience, PS was indicated for limited staff resources during the COVID-19 pandemic to treat five non-deferable cases. The sixth and seventh patients were selected for PS as they already had a functioning axillo-bifemoral bypass that was used for this purpose. For the most recent cases, PS was chosen as the primary perfusion method according to a score based on clinical and anatomical factors with ECC as a bailout strategy. Selective renal perfusion with cold (4 °C) Custodiol solution was the method of choice for renal protection in all cases while antegrade perfusion of the coeliac trunk and the superior mesenteric artery was assured by PS through a loop graft (8–10mm) proximally anastomosed to the axillary artery (10 patients, 90.9%) or the descending thoracic aorta (one patient, 9%) and distally anastomosed to the infrarenal aorta (3), common iliac (3), or femoral vessels (5). In-hospital mortality was 9% as one patient died on the 10th postoperative day from mesenteric ischemia following hemodynamic instability; permanent spinal cord ischemia rate was 0% and the rate of AKI stage 3 was 9% (one patient). Bailout shifting to ECC was never required. No cardiac complications, nor a significant increase in serum CK-MB were reported in any patient. No prolonged severe intraoperative hypotension episodes (Mean Arterial Pressure < 50 mmHg) were assessed using the Software Acumen Analytics (Edwards LifeSciences, Irvine CA, USA). No peri-operative coagulopathy nor major bleeding was reported. **Conclusions**: Our experience showed satisfactory outcomes with the use of PS in specifically selected cases. Current data indicate that PS may represent an alternative to ECC techniques during TAAAs OR in high volume centers where assisted extracorporeal circulation could eventually be applied as a bailout strategy. However, due to the small sample size of this and previously published series, more data are needed to clearly define the potential role of such approach during TAAA OR.

## 1. Introduction

In the treatment of thoracoabdominal aortic aneurysms (TAAAs) Fenestrated and Branched Endovascular Repair (F/BEVAR) is routinely used in unfit patients whenever anatomically suitable and has progressively been extended also to younger patients, despite open surgery still represents the standard of care [1,2].

Indeed, previous data indicate that endovascular repair is associated with improved early outcomes but results from the high volume center show that open TAAA repair guarantee acceptable short-term outcomes with better intermediate-term results than F/BEVAR: in matched patients, OR achieved similar in-hospital death (8.3% vs. 7.6%, *p* = 0.80) and neurological complications (3.6% vs. 2.2%, *p* = 0.30), and improved 10-year survival than endovascular repair (52% vs. 33%, *p* < 0.0001) with a lower need of aortic reintervention (4% vs. 21%, *p* < 0.0001) [3].

Nevertheless, open repair (OR) of TAAA still presents a complex set of challenges that demand a delicate balance between a life-saving intervention and potentially severe complications. During the past decades, new techniques and devices have been introduced to optimize the operative strategy, but the complex management of the massive hemodynamic changes intraoperatively, and the perfusion of viscera, kidney, and spinal cord remain the main concerns [1].

Indeed, proximal aortic cross-clamping leads to a significant increase in left ventricular afterload, which could potentially induce myocardial dysfunction and cerebrovascular complications [1]. Furthermore, the need to interrupt the inline aortic blood flow during surgery questions the optimal strategy to prevent visceral, peripheral, and spinal cord ischemia, which leads to increased mortality and morbidity [1].

Several techniques have been developed over time to prevent the fearful consequences of proximal aortic cross-clamping. At the beginning of the history of TAAA OR, passive shunting (PS) has been used to bypass the descending thoracic aorta (DTA) excluded from circulation, allowing for distal perfusion [4,5,6].

Its application had a short life, as PS was soon replaced by the more innovative and replicable techniques of assisted extracorporeal circulation (ECC). Indeed, the widespread diffusion of ECC such as cardiopulmonary bypass (CPB) and left heart bypass (LHB) for cardiothoracic operation has ensured standardized, reliable, and predictable flow for visceral and continuous distal perfusion [7,8,9,10]. In contrast, several methods for spinal cord protection (i.e., cerebrospinal fluid drainage, systemic hypothermia, and selective perfusion of intercostal vessels) have shown promising results [11,12,13,14,15].

However, thanks to its potential to mitigate some of the drawbacks of assisted extracorporeal circulation, PS has never been completely abandoned, and a few centers have continued to employ this technique in selected patients [16,17,18].

In this scenario, we describe our institutional bundle for the use of PS during TAAAs OR. We aimed to investigate the results achieved during ours and previous experience. A comprehensive literature review exploring the use and applicability and the reported clinical results (visceral, renal, and spinal cord complications and mortality of PS during TAAAs OR was therefore also performed.

## 2. Materials and Methods

All consecutive patients who underwent OR for TAAA between March 2021 and December 2023 at our institution were entered into a prospectively maintained electronic database that was then retrospectively reviewed.

A cohort of patients operated on using PS was identified and included in the present analysis with the aim to investigate the rate of in-hospital mortality, and renal (according to the KDIGO, Kidney Disease Improving Global Outcomes guidelines), visceral (bowel ischemia not resolving with medical therapy or requiring surgical resection), and spinal cord ischemia achieved in this setting [19,20].

Visceral ischemia was defined as the combination of laboratory (elevated serum lactate and/or liver function test levels) and imaging (lack of opacification of the mesenteric vessels or signs of gut infarction).

SCI was defined as any new motor or sensory deficits of the lower extremity in the absence of documented intracerebral hemispheric events.

Acute renal failure was described as a sudden reduction in kidney function, as measured by increased creatinine and/or decreased urine volume.

The potential advantages and drawbacks of PS, as reported in previous series, were also investigated in a comprehensive literature review. The search was performed based on three databases (PubMed, EMBASE, and Cochrane Library) by two independent reviewers (LS and AA) from inception to 31 December 2023 and the reported clinical results (visceral, renal, and spinal cord complications and mortality) using PS during TAAAs OR were analyzed.

### 2.1. Institutional Protocol for the Management of Thoracoabdominal Aortic Disease

At our institution, patients affected with TAAA are addressed to either open or endovascular repair with F/BEVAR only following full assessment of their fitness for surgery (using ASA core, frailty screening test in patients older than 70 years, transthoracic echocardiography, TEE, and stress capacity imaging) and according to the aneurysm’s anatomy.

A multi-systemic preoperative assessment is routinely performed and a preoperative Computed Tomography Angiography (CTA) of the whole aorta is always postprocessed with Aquarius workstation (TeraRecon Inc., Foster City, CA, USA) to investigate the size, extent, and morphology of the TAAA and the status of collateral vessels and evaluated by a consultant radiologist and vascular surgeon.

When OR is indicated, tissular oximetry by Near InfraRed Spectroscopy (NIRS) and Motor Evoked Potential (MEP) are systematically used during the intervention. CerebroSpinal Fluid Drainage (CSFD) with the LiquoGuard (Möller Medical GmbH, Fulda Germany) system is routinely inserted for Crawford II and III TAAAs due to the increased risk of SCI reported in these patients.

Postoperative CFSD is performed in the other cases (with less extensive disease and the need of more distal application of aortic cross-clamping) if neurological symptoms occur, to maintain pressure below 10 cm H_2_O in association with permissive hypertension (Mean Arterial Pressure 80–90 mmHg), optimization of hemoglobin level (>10 g/dL), glycemia, and oxygen saturation. During the immediate postoperative period, a sedation hold is performed after 2–3 h aiming to assess lower limb mobility in all patients guiding clinical decision on CFSD managing or insertion not having undergone prophylactic placement.

Operative technique for TAAA OR has already been extensively described elsewhere, but specificities of our surgical approach are as follows:

- Use of sequential aortic cross-clamping;
- Use of blood perfusion for the celiac trunk and superior mesenteric artery and cold (4 °C) perfusion with Custodiol solution (Custodiol; Dr Franz-Kohler Chemie GmbH, Bensheim, Germany) for renal arteries;
- Separate reattachment of visceral and renal arteries when anatomically feasible;
- Reimplantation of at least two patent intercostal arteries whenever doable.

The use of systemic heparinization was standardized for all patients (1 mg/kg) and checked intraoperatively (measuring Activated clotting time -ACT with a target of 250–300). A cell-saving device (Cell Saver 5TM, Haemonetics, Braintree, MA, USA) was used in all cases.

Postoperatively, before extubating, prophylactic bronchoscopy is performed, and chest physiotherapy is always started from postoperative day one to improve respiratory status in all patients.

All patients regularly undergo biological monitoring of visceral and renal ischemia during the Intensive Care Unit (ICU) and ward stay and imaging is performed based on clinical or biological evidence of ischemic complications for the kidney, bowel, or spinal cord.

At the discharge, a regular clinical and CTA surveillance is planned at 1-, 6-, and 12-month intervals, followed by annual control thereafter.

### 2.2. Passive Shunting: Indication

At our institution, TAAA surgery is routinely performed under ECC.

In our early experience, PS was indicated for limited staff resources during the COVID-19 pandemic to treat five non-deferable cases (one symptomatic patient treated on emergent basis and four patients presenting with rapidly growing TAAA). The sixth and seventh patients were selected for PS as they already had a functioning axillo-bifemoral bypass that was used for this purpose.

Based on the satisfactory results achieved with these cases, we developed a score for PS eligibility based on clinical and anatomical factors as follows:

*TAAA anatomy*

- Dissecting aneurysm: Yes 0 point; No 1 point;
- TAAA extent: Type II 0 point; Type II with clamping site distal to LSA: 1 point; Type I or V: 2 points; Type III: 3 points; Type IV: 4 points;

*Cardiac status*

- Left Ventricle Ejection Fraction (LVEF) <30%: 0 point; >50%: 1 point; >60%: 2 points;
- No valvular disease: 1 point;
- No atrial fibrillation: 1 point;
- Sizes of cardiac chamber within the normal range: 1 point;
- Preoperative derivative of pressure over time (dP/dt) > 500: 1 point.

*Comorbidities*

- Infection: 2 points;
- Cancer: 2 points;
- Contraindication to systemic heparinization: 1 point.

*Prior bypasses*

- Prior femoro-femoral cross-over bypass: 1 point;
- Prior axillofemoral bypass: 2 points.

Only patients presenting with a score > 8 were considered eligible for this strategy following discussion in a multidisciplinary team for reviewing of imaging and clinical review.

### 2.3. Passive Shunting: Surgical Technique

**Figure 1:**
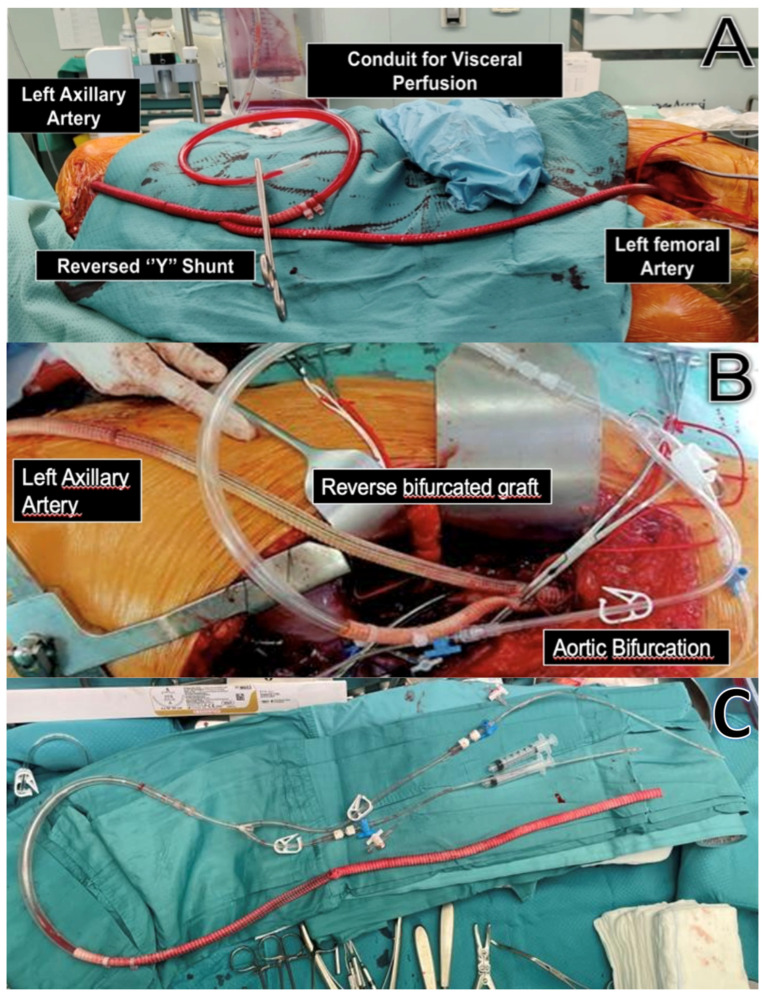
Intraoperative images of a passive shunt. (**A**): A passive shunt between the LAA and the LFA was created using an 8 mm Dacron prosthesis to provide blood flow for visceral perfusion during aortic cross-clamping through a side branch. (**B**): The picture shows a different configuration of PS in which an external bypass is created between the left axillary artery and the infrarenal aorta using a reversed bifurcated 8 mm Dacron graft. (**C**): Elemental circuit for PS: a 8 mm Dacron graft is connected with a “Y-bifurcated” line with two 9F Pruitt occlusion-perfusion balloon catheters for visceral vessels perfusion. The other end of the prosthetic graft will be anastomosed to an inflow vessel to assure perfusion during aortic cross-clamping.

**Figure 2:**
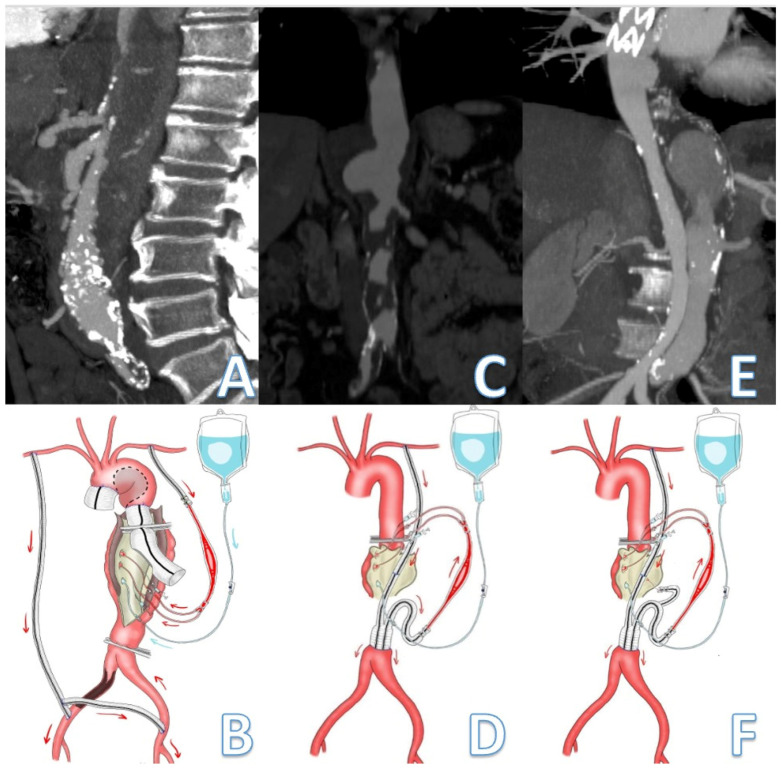
**Schematic representation passive shunt according to the extension of the thoracoabdominal aortic aneurysm.** Visceral and lower limb perfusion is assured by the passive shunt while Custodiol solution is used for renal arteries perfusion. A patient presenting with a type II dissecting TAAA. (**A**,**B**) Preexisting axillo-bifemoral bypass was used to perfuse the lower limbs while a second graft anastomosed to the LSA provided flow to the visceral vessels. (**C**,**D**) A PS between the LSA and the infrarenal aorta was created using a reversed bifurcated 8 mm Dacron graft for visceral perfusion during treatment of a type III TAAA. (**E**,**F**) A PS between the LSA and infrarenal aorta assure revascularization of CT, SMA, and intercostal artery during surgical repair of a type II-dissecting TAAA in a patient who has previously undergone TEVAR. The arrows indicate the direction of the blood flow from the inflow vessel to assure visceral perfusion during aortic cross-clamping.

**Figure 3:**
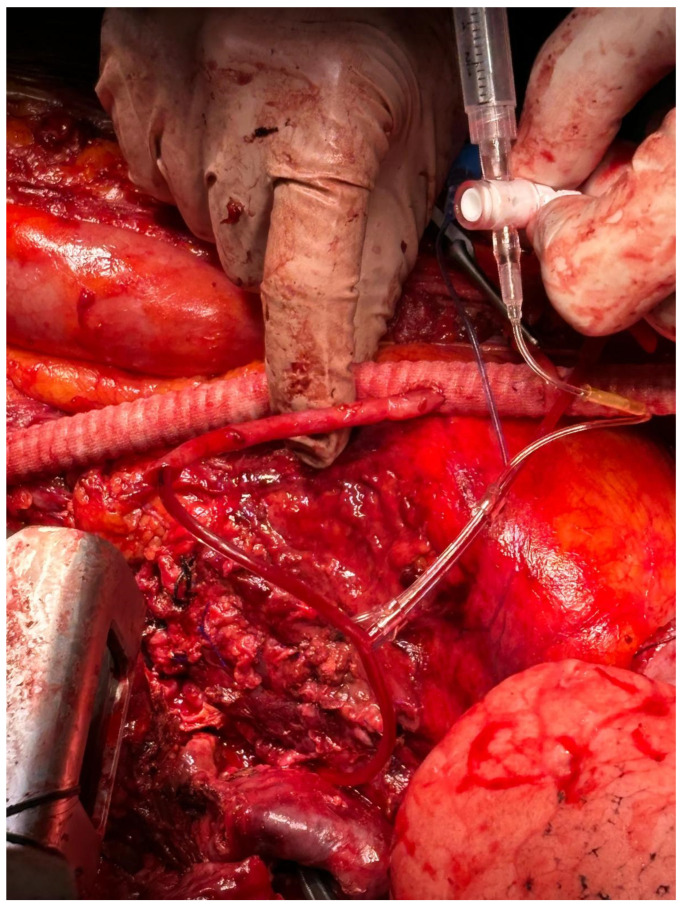
**Intraoperative details of a passive shunt.** A passive shunt from the axillary to the infrarenal aorta is used for perfusion of the superior mesenteric artery. A saphenous vein graft was sutured to the PS and cannulated with the Pruitt shunt to allow subsequent aorto-mesenteric bypass.

Passive shunting is manufactured with an 8–10 mm Dacron prothesis at the end of which is positioned a “Y-bifurcated” line with two 9F Pruitt occlusion-perfusion balloon catheters. The Pruitt catheters are inserted directly into the celiac and superior mesenteric arteries for antegrade selective perfusion (Figure 1, Figure 2 and Figure 3).

In this series, the inflow was assured either by the left axillary artery (10 cases) or the descending thoracic aorta (DTA) (one patient) while distal outflow was the infrarenal aorta (3 cases), common iliac (3 cases), or common femoral arteries (5 cases) according to the TAAA extent and quality (presence of calcifications, thrombus, or vessels angulation) of the abdominal and inguinal vessels. LSA was considered as the preferential inflow site as it allows for the instauration of the PS even before the dissection of DTA preventing potential low flow to the visceral vessels in case of emergency or need for more proximal aortic cross-clamping.

### 2.4. Intraoperative Monitoring

**Figure 4:**
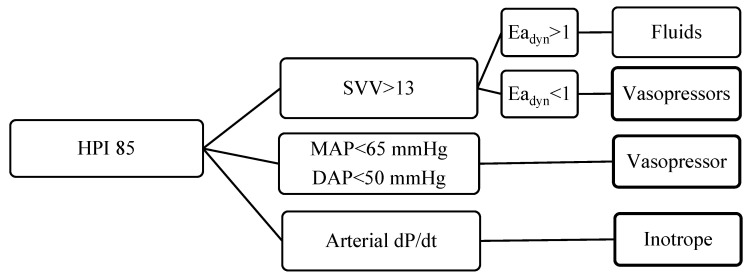
Predictive algorithm applied to arterial hypotension and hemodynamic instability.

For all patients, a preoperative radial arterial cannula was placed (20G, Vygon) and connected to the advanced monitoring platform Hemosphere (Edwards LifeSciences, Irvine, CA, USA). A predictive algorithm (Figure 4) was adopted by using the Hypotension Prediction Index (HPI), to avoid arterial hypotension (Mean Arterial Pressure, MAP > 65 mmHg) and hemodynamic instability. The Hemosphere platform was used continuously throughout the procedure, and the anesthesiologist was responsible for interpreting the data in real time.

Hypotension Prediction Index (HPI), Mean Arterial Pressure (MAP); Diastolic Arterial Pressure (DAP); dynamic arterial elastance (Ea dyn); stroke volume variation (SVV); dP/dt derivative of pressure over time (dP/dt).

The trigger for any intervention was the HPI value of 85; then a decision tree was represented in a secondary screen according the following algorithm: in case of fluid responsiveness (i.e., SVV > 13) a bolus of 100 mL of crystalloids was adopted in case of low vascular compliance (Ea_dyn_ > 1); with SVV > 13 and Ea_dyn_ < 110 mcg of norepinephrine was administered. This drug was also given in case of non-fluid responsiveness (SVV < 13 with MAP < 65 mmHg and/or DAP < 50 mmHg). The inotropic agent ephedrine was administered in case of arterial dP/dt < 400.

The foresight sensors were used and bilaterally placed at the level of the calf and flank to observe the hemodynamic changes and tissue saturation response to the clamping and de-clamping.

The decision to shift to CEC was based on the response of the pressure to time ratio (dP/dt), which represents a surrogate of the contractility during the aortic clamping with a cut-off of 400 according to previously published experiences.

### 2.5. Ethical Statement

This study was performed in accordance with the Institutional Ethical Committee rules. All included patients provided consent for intervention with the use of PS and/or ECC. As it was a retrospective review for service evaluation, within an audit approved by our Surgical Department, and there was no prospective randomized study nor modification in patient’s care but application of already described treatment strategies, the final ethical approval of our Institutional Ethical Committee was not required.

## 3. Results

### 3.1. Single-Center Experience

Between March 2021 and December 2023, 51 TAAAs repairs were performed, and 11 patients were operated using PS. Included patients were mostly male (8, 73%) and their mean age was 67 years (range 63–79). Patients’ demographics and comorbidities are summarized in Table 1.

All patients were affected with hypertension (11, 100%) and most had dyslipidemia (9, 82%) and a history of tobacco dependence (7, 64%). None of the included patients had a collagenous disease as demonstrated through genetic testing performed pre- or postoperatively according to age, clinical signs, and family history.

Indications for surgery were atherosclerotic aneurysms (9) or chronic type B aneurysmal dissections (2). The aneurysm’s extent according to the Crawford classification was type I in one (9%) patient, type II in another one (9%), type III in two (18%) cases, type IV in five (45%), and type V in the remaining two (18%) patients. Anatomical data and configuration of PS are reported in Table 2.

### 3.2. Intraoperative Outcomes

All patients (100%) received vasopressor, as norepinephrine infusion immediately after the induction was started and in two patients (18%) an administration of the inotropic agent dobutamine was required based on the low value of the arterial dP/dt.

All patients (100%) experienced arterial hypotension after the administration of propofol, which lasted less than 30 s and counterbalanced with etilephrine administration.

The median monitoring time for patient was 408 min, with a 3 to 5 of hypotensive events for patient. The average duration of each hypotensive event was 1.47 ± 1.04 min with no related clinical impact. The Time Weighted Average of Area under thresholds (TWA-MAP < 65 mmHg) was 0.24 mmHg. No patients had severe arterial hypotension (MAP < 50 mmHg).

**Figure 5:**
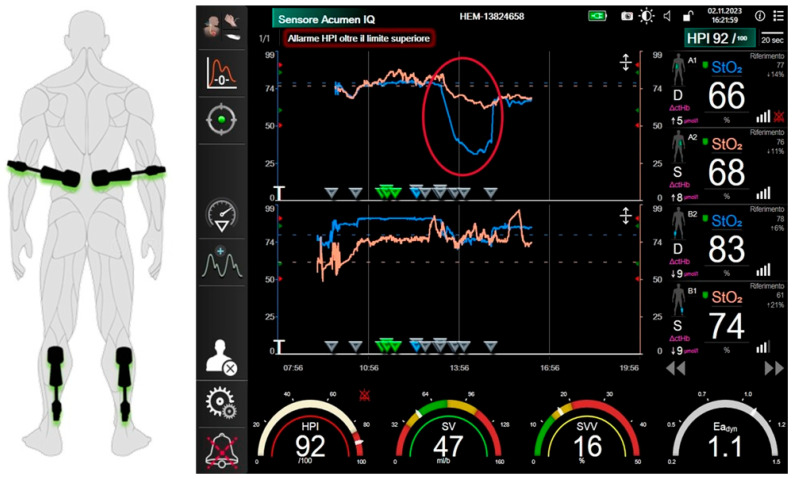
Monitoring of hemodynamic changes and tissue saturation response to the aortic cross-clamping. (**A**): Positioning of the foresight sensors. (**B**): Collapse of lumbar perfusion mainly at the right side during aortic cross-clamping probably due to the Sicard positioning.

No cardiac complications, nor a significant increase in serum CK-MB, was reported in any patient. Interestingly was the effect of the aorta cross-clamping on the tissue saturation (StO2); we observed in all patients a drop of the StO2 in both the sensors placed at the level of the calves and at the level of the flank only in the sensor placed in the right side probably due to the Sicard position for which no treatment adjustment was deemed necessary as no clinical impact was noted (Figure 5).

### 3.3. Postoperative Outcomes

Peri-operatively, no cases of cardiac complications, coagulopathy, or significant bleeding (significant drop in hemoglobin (≥2 g/dL) or requirement for multiple blood transfusions) necessitating reintervention were reported.

Pulmonary complications were observed in two patients (18%): one patient experienced a pneumothorax that was treated with a thoracic drainage, whilst the other developed pleural effusion and respiratory failure requiring percutaneous drainage and re-intubation (Table 3).

Transitory paraplegia (motor deficit L3-S1 grade 3, sensory deficit L5-S3 grade 1–2) [20] occurred on postoperative day 1 in one patient (9%) who had undergone extensive IV repair. He fully recovered following CSFD insertion.

Postoperative stage 3 acute kidney injury (AKI) occurred in one patient (9%) with a preoperative estimated glomerular filtration rate (eGFR) of 41 mL/min/1.73 m^2^. He needed hemodialysis until death as detailed below. Two other patients, without any previous history of kidney disease, experienced stage 1 AKI that was successfully managed with medical treatment and completely regressed before discharge. No complications related to the inflow or outflow vessels were reported in any case.

Mean postoperative Intensive Care Unit (ICU) length of stay was 5 days (range 1–10), and mean hospital stay was 14 days (range 9–20).

At a mean follow-up of 18 months (range 10–42 months), the overall mortality was 18% (2 cases): one patient (9%) with a history of CAD died in-hospital from mesenteric ischemia with intestinal perforation and multiorgan failure (MOF) on the 10th postoperative day following hemodynamic instability due to cardiac failure requiring maximum norepinephrine and inotropes doses; another patient (9%) presenting with a post dissection type II TAAA and treated several years previously with an ascending aorta replacement died 15 months after surgery from a suspected septic rupture of an anastomotic pseudoaneurysm developed on the ascending graft.

## 4. Discussion

Changes in blood perfusion to the spinal cord, kidneys, and viscera significantly contribute to the development of ischemic complications during aortic cross-clamping for TAAA OR so that, in recent decades, several strategies for organ protection and distal perfusions have been developed.

The need of aortic cross-clamping more proximal than LSA mandates the use of cardiopulmonary bypass (CPB) with or without deep hypothermic circulatory arrest (DHCA) and antegrade cerebral perfusion (ACP) to minimize visceral and lower body ischemia. Alternate techniques consist of the use of left heart bypass (LHBP) and distal aortic cannulation, cerebrospinal fluid drainage, and selective visceral branch perfusion strategies.

Among those, PS represents a historical strategy that has been used in the treatment of DTA pathologies and more rarely during aortic arch surgery. The literature about the applicability of PS during TAAA OR has been relatively poor over the last 30 years [6,16,17,18].

Indeed, our literature search generated 66 articles, four of which were included after full-text reading, totaling 64 patients [6,16,17,18]. All included studies were retrospective cohorts, and none compared PS to other active shunting methods or to aortic cross-clamping without any distal perfusion strategy (Table 4). 

Different configurations of PS were described: Comerota et al. [6] used a temporary axillofemoral bypass in treatment of 15 TAAAs (4 type I, 3 type II, 5 type III, 3 type IV); Monnot et al. [16] used a temporary axillary bypass to the superior mesenteric and right renal artery in 10 type III TAAAs, while left renal artery was perfused with 4 °C Lactate Ringer solution; Cambria et al. [17] focused only on visceral protection, with celiac trunk or superior mesenteric artery cannulation from a passive shunt originating from the aortic prosthesis (15 patients: 5 type I, 6 type II, 4 type III TAAAs) after completion of the proximal anastomosis; finally, Bouziane et al. [18] reported 24 cases of type IV TAAAs in which PS from the DTA was used to perfuse the superior mesenteric artery and both renal arteries.

Most of patients were electively treated (55, 86%) while one patient (1.6%) underwent urgent repair and eight (12.5%) were operated in an emergent setting. Authors decided to use PS with the aim to decrease the major hemodynamic disturbances that accompany TAA repair including cardiac afterload, spinal cord ischemia risk by avoiding cephalic hypertension and caudal hypotension, and avoiding hypotension during declamping and the need for heparin [6,17,18].

Monnot and colleagues conversely used PS in a selected group of patients affected with TAAA and severe iliac occlusive disease stating that distal retrograde perfusion is ineffective for visceral protection in such scenarios [18].

No cases of mesenteric ischemia, peri-operative coagulopathy, or major bleeding were reported. Overall mortality was 3% (2/64) and the postoperative AKI rate was 7.8% (5/64), but persistent renal insufficiency presented in only 1.5% (1/64) of patients at the discharge.

Although one study did not disclose the rate of neurological complications [18], overall paraplegia was reported to occur in 2% (1/49) of cases.

Due to the variability, the low number of cases and incomplete data with possibly underreporting of complications, it was not possible to perform pooled statistical analysis or meta-analysis.

Additionally, despite the initial promising results, PS has been progressively replaced by active ECC methods that have become the standard of care in high-volume aortic centers as this allows for a standardized and predictable flow for bowel and kidney that do not relay on the hemodynamic status of the patient preventing low flow also in case of massive hemorrhage or cardiac failure [7,8,9,10,21,22].

DHCA is mandatory when proximal aortic cross-clamping is unfeasible due to thrombus, significant aortic calcifications, or adhesions or porcelain aorta, but overall, ECC techniques (CPB and LHBP) currently represent the optimal strategy and are particularly useful when major intraoperative blood losses are expected (i.e., during treatment of dissecting TAAAs with wide patent false lumen), or in the treatment of Crawford I and II TAAAs that require an aortic cross-clamping proximally to the LSA, leading to a massive increase in ventricular afterload [23,24]. LHBP provides some benefit over CPB as it requires lower levels of heparinization and offers cardiac protection by reducing the afterload during the aortic cross-clamping.

PS still has more potential in mitigating some of the drawbacks of the use of extracorporeal circuits connected with centrifugal or roller pumps such as the need for cardiac and great vessels cannulation, priming and large intravascular fluid volume shifts, and use of high doses of heparin.

Actually, ECC techniques are linked to a severe postoperative coagulopathy, vasoplegia, immunosuppressive effects, an increase in oxidative stress markers, and systemic inflammatory response syndrome (SIRS) related to the exposure of blood to foreign material and non-endothelial circuit compound (e.g., tubing, oxygenator) potentially leading to renal, pulmonary, and neurological complications, and increased risk of bleeding or even multiple organs dysfunction [25,26,27,28]. No quantitative comparative analysis could be performed in the light of current evidence, but this is by definition avoided with PS that do not require extra-corporeal circulation. This is corroborated by previous findings on off-pump coronary heart bypass.

Indeed, in that regard, several authors have already reported about the advantages of avoiding ECC during myocardial revascularization so that off-pump coronary artery bypass grafting (OPCAB) and minimally invasive direct coronary artery bypass (MIDCAB) have gained widespread diffusion as alternative techniques to conventional on-pump coronary artery bypass grafting (CABG) [29].

On the other hand, historical series reported promising results with the use of PS despite the heterogeneity and paucity of cases represent an inherent limitation and the lack of randomized controlled trial limit the power of the analysis, the mortality rate was satisfactory (0–9% versus 6.9–11.7% using standard ECC techniques) regardless of the aneurysm’s extent, and the rates of complications for the spinal cord (temporary SCI rate 2–9% versus 3.6–5%) and kidneys (7.8–9% versus 7.9%) were promising [6,16,17,18,30,31].

No comparative studies on humans have been conducted so far, but Liu and colleagues compared in an animal model the use of no pump, passive shunting (between the left common carotid and left femoral arteries), and centrifugal pump (LHBP) during aortic cross-clamping of the descending thoracic aorta. The authors demonstrated that all animals in the group without perfusion suffered from hemodynamic instability, metabolic abnormal, and neurologic injury and died within 12 h. Conversely passive shunt was associated with lesser hemodynamic instability and neurological sufferance than active pumping, leading the authors to conclude that passive shunting is superior to active shunting for aortic bypass [32].

In this setting, we believe PS may regain interest among surgeons for some potential advantages:

- It allows blood to flow directly from the aorta or one of its branches (e.g., the axillary artery) into the visceral and spinal arteries during aortic clamping, reducing left ventricular afterload and thereby limiting proximal hypertension.
- It avoids recirculation of contaminated blood (e.g., in case of preoperative coagulopathies, cancer, mycotic aortic aneurysm, or infection) and risk of SIRS form contact with tubing.
- Thanks to the intuitive functioning and handiness, it could be considered in urgent settings even in peripheral centers where ECC systems and a perfusionist team are not routinely available.

Beyond these theoretical advantages, the dose of heparin needed during TAAA OR with PS is much less (ACT target of 250–300 or even a single dose of 50UI/Kg) [16] than that commonly used during cardiopulmonary bypasses (as per current international guidelines an ACT value of >480 s is considered an appropriate threshold) [33] limiting the risk of bleeding.

Additionally, PS provides direct pulsatile flow to the abdominal organs that has shown to be a factor associated with lower rate of acute renal insufficiency, lower values of arterial blood lactates postoperatively, shorter intubation time, and shorter stays in the ICU and hospital in a meta-analysis of randomized controlled trials (RCTs) analyzing pulsatile versus continuous flow during ECC [30].

Anyhow, we acknowledge some limitation of such strategies: during CPB cardiocirculatory and respiratory functions are completely replaced by the heart–lung machine, allowing rapid reinfusion of massive blood losses with a well-defined and predetermined flow to the distal aorta and visceral vessels that is set on Body Surface Area (BSA) and temperature (under moderate hypothermia-to-normothermic conditions, the pump flow rate is normally set between 2.2 and 2.8 L/min/m^2^) [30]. Patients included in this, and previous studies did not experience an increased rate of ischemic complications for the spinal cord, viscera, and kidneys, but measuring systemic blood flow or tailoring perfusion to the target vessels using PS is basically impossible as it allows perfusion only based on systemic arterial blood pressure and conduits’ diameters.

In that regard, several factors (metabolic status, body temperature, and BMI) variously impact on actual bloods needs during TAAA OR but the size of the cannula should be theoretically selected based on patient size and weight, anticipating the theoretical flow rate to the vessel to be cannulated and also considering the catheter flow volume and resistance [34,35] so that further studies with animal model evaluating intestinal ischemia–reperfusion injury with histopathologic changes evaluation according to cross-clamping levels and using different cannula sizes and perfusion flow rate are indicated [36].

No evidences currently at disposal on this matter, but previous experience with retrograde perfusion from the infrarenal aorta of visceral and renal arteries during cross-clamping even suggested using 12–16 Fr catheters assuring a targeted blood flow of >300 mL/min through each of the selective catheters [37,38,39]. However, since the estimated volume of blood to the superior mesenteric artery in fasting individuals have been estimated to range between 639 +/− 153 mL/min, our standard protocol for visceral perfusion is to use 9 Fr catheter for the superior mesenteric and coeliac artery according to previously published series on visceral and renal perfusion during TAAA OR [38,39,40,41,42]. This approach in this series demonstrated to provide adequate flow during cross-clamping with no reported cases of visceral ischemia.

Overall, considering our historical results, we believe that PS may be applied in selected cases during TAAA OR in high volume centers where assisted ECC could eventually be used as a bailout strategy. Although more robust data on its performance over ECC are needed to suggest a more extensive application of such a strategy, the preliminary results achieved using our protocol for selecting eligible patients for PS suggest that a good cardiac function preoperatively and need for less extensive repair (Type III and IV TAAA) are mandatory prerequisite of successful TAAA OR without ECC techniques.

## 5. Conclusions

This represents a preliminary experience with the use of PS in a highly selected group of patients.

In this series, the use of PS during TAAA OR was associated with satisfactory clinical results in the short- to medium-term. When specific anatomical and clinical aspects are fulfilled, in high volume centers and with ECC at disposal as a bailout strategy, PS could represent a potential option requiring further study.

## 6. Limitation

This study has several limitations: the small number of patients restricts statistical analyses in terms of significance, and the sample size is too small for drawing strong conclusions. Additionally, there was no direct comparison group with other perfusion strategies, and the follow-up was relatively short.

Also the literature review has several limitations as the number of available studies was small, with limited sample sizes and no randomized controlled trials and significant heterogeneity was noted in terms of patients’ selection and outcomes reporting and lack of control groups.

The reported results should be interpreted with caution and underscore the need of larger studies to better define the applicability and potentiality of PS during TAAA OR.

## Figures and Tables

**Table 1:** Patients’ demographics and comorbidities.

Patients	Total (n/%)
Sex	
Male	8 (73.6)
Female	4 (36.4)
Mean Age	67 years (range 63–79)
TAAA Extent (Crawford Classification)	
Type I	1 (9)
Type II	2 (18.2)
Type III	-
Type IV	6 (54.5)
Type V	2 (18.2)
Comorbidities	
Hypertension	11 (100)
Dyslipidemia	9 (82)
Diabetes mellitus	-
CAD	3 (27.3)
COPD	2 (18.2)
Chronic kidney disease	5 (45.5)
Tobacco use	7 (63.7)
ASA class	
1	-
2	2 (18.2)
3	9 (82)
4	-
Clinical presentation	
Asymptomatic	10 (91)
Thoracic Pain	1 (9)

CAD—Coronary Artery Disease; COPD—Chronic Obstructive Pulmonary Disease; ASA—American Society of Anesthesiologists.

**Table 2:** Anatomical data and configuration of passive shunt.

Patient (Sex/Age)	TAAA Type *	Previous Aortic Surgery	PS Inflow	PS Outflow	Morbidity	Mortality	Preoperative Score ^#^
1 (M—72 years)	IV	AA replacement	LAA	LCIA	-	No	9
2 (M—65 years)	IV	Infrarenal aortic surgical repair	LAA	Infrarenal aorta	AKI	No	8
3 (M—66 years)	IV	-	LAA	LCIA	AKI, SCI, mesenteric ischemia	Yes	8
4 (F—72 years)	I	TEVAR	LAA	LCIA	-	No	8
5 (F—51 years)	III	AA replacement	LAA	Infrarenal aorta	-	No	9
6 (M—63 years)	II	AA replacement + right axillofemoral and femoro-femoral bypass	LAA	RCFA	-	Yes	10
7 (M—66 years)	IV	Axillofemoral	LAA	LCIA	-	No	10
8 (M—78 years)	III	EVAR	LAA	RCIA	AKI	No	8
9 (M—69 years)	V	-	LAA	Infrarenal aorta	-	No	8
10 (F—67 years)	IV	-	LAA	LCFA	-	No	9
11 (M—69 years)	V	AA replacement; TEVAR	DTA	LCIA	-	No	8

AA—Ascending Aorta; LAA—Left Axillary Artery; LCFA—Left Common Femoral Artery; RCFA—Right Common Femoral Artery; LCIA—Left Common Iliac Artery; RCIA—Right Common Iliac Artery; EVAR—EndoVascular Aortic Repair; TEVAR—Thoracic EndoVascular Aortic Repair; DTA—Descending Thoracic Aorta; AKI—Acute Kidney Injury; SCI—Spinal Cord Ischemia. * According to Crawford classification. ^#^ According to our institutional bundle. Ten patients (90.9%), four of which presented a rapidly growing TAAA, were asymptomatic and electively treated; the remaining one presented with thoracic pain (9%) and underwent urgent intervention.

**Table 3:** Operative details and postoperative outcomes.

*Operative Details*	*Total (n/%)*
**Setting**	
Urgent/emergent	1 (9)
Elective	10 (91)
**Cerebrospinal fluid drainage**	
Preoperative insertion	4 (36.4)
Postoperative insertion	1 (9)
***Outcomes***	***Total (n/%)***
**In-hospital mortality**	1 (9)
**Mortality during follow-up**	1 (9)
**Paraplegia**	
Transitory	1 (9)
Permanent	-
**Renal failure**	
Transitory	2 (18.2)
Permanent	1 (9)
**Mesenteric ischemia**	1 (9)
**Bleeding requiring reoperations**	-
**Mean ICU stay**	5 days (range 1–10)
**Mean hospital stays**	14 days (range 9–20)
**Mean follow-up**	18 months (range 10–42)
*ICU Intensive Care Unit*	

**Table 4:** Literature review.

Author, Year	N Patient/Aneurysm Extent	Type of PS	Setting/Rationale for PS Use	Arteries Perfused with PS	Postoperative AKI	AKI at the Discharge	SCI	Peri-Operative Coagulopathy Major Bleeding Mesenteric Ischemia	Mortality
Comerota et al. [6] 1995	15 TAAA (4 type I, 3 type II, 5 type III, 3 type IV)	Axillofemoral bypass	Ps was used reduce the cardiac afterload of thoracic aortic clamping		1 (7%)	0 (0%)	1 (7%)	-	1 (7%)
Monnot et al. [16] 2016	10 type III TAAA	Temporary axillary bypass	Severe aortoiliac occlusive disease that would have limited retrograde perfuse to visceral vessels during proximal aortic anastomosis	Both SMA and RRA (LRA perfused with 4 °C Lactate Ringer solution)	1 (10%)	1 (10%)	-	-	-
Cambria et al. [17] 1998	(15 patients: 5 type I, 6 type II, 4 type III TAA)	Aortic prosthetic body	PS was used to reduce metabolic and hemodynamic derangements occurring with reestablishment of visceral perfusion during clamp and sew TAA repair	CT or SMA	-	-	-	-	-
Bouziane et al. [18] 2018	24 cases of type IV TAAA		NR	SMA, RRA, LRA	3 (12.5)	-	NA	-	1(4%)

PS—Passive Shunt; AKI—Acute Kidney Injury; SCI—Spinal Cord Ischemia; TAAA—Thoracoabdominal Aortic Aneurysm; SMA—Superior Mesenteric Artery; CT—Coeliac Trunk; RRA—Right Renal Artery; LRA—Left Renal Artery; NA—Not Applicable; NR—Non-Reported.

## Data Availability

The raw data supporting the conclusions of this article will be made available by the authors on request.

## Abbreviations

The following abbreviations are used in this manuscript:

ICU	Intensive Unit Care
FU	Follow-Up
SD	Standard Deviation
ASA	American Society Anaesthesiology
CKD	Chromic Kidney Disease
AAA	Abdominal Aortic Aneurysm
AR	Amputation Rate
DUS	Doppler Ultrasound
DVT	Deep Vein Thrombosis
CTA	Computed Tomography Angiography
PAAs	Popliteal Artery Aneurysms
OR	Open Repair
ER	Endovascular Repair
